# The brain–the organ of the psychic (The lesions / the defense mechanisms)

**Published:** 2010-08-25

**Authors:** V Rotarescu, AV Ciurea

**Affiliations:** * >Department of Neurosurgical ‘Bagdasar – Arseni’ Clinical Hospital, ‘Carol Davila’ University of Medicine and Pharmacy Bucharest Romania; ** >Neuropsychological Laboratory of ‘Bagdasar–Arseni’ Clinical HospitalRomania

## Abstract

The article is based on the Leopold Szondi theory (March 11, 1893 – January 24, 1986), who was a Hungarian psychiatrist. He is known for the psychological tool that bears his name, the Szondi test. He developed a form of depth psychology that had some prominence in Europe in the mid–20th century, but has been ignored for the most part), the study seeks to correlate the szondian test results with the imagistic ones on a wide–range pathology. In the Neurosurgery Department, patients are investigated using modern exploration methods (MRI, CTscan, and computed EEG, etc.) in order to identify possible somatic lesions.

The study's subjects selected during 2000–2004 from the patients admitted and investigated for neurosurgical conditions; they were divided into two subgroups, based on whether the organic lesions were or were not present (the independent variable). The exclusion criterion was a lesion due to external causes. Statistically meaningful there are seven types of Ego profiles, in relation with the lesion: the archaic ego [0 –], the inhibited ego [– +], the adaptive ego [– –], the narcissist ego [+ +], the identified ego [+/– 0], the fugitive ego [+/– –] and the possessed ego [0 +]. The nexus in the destiny's analysis description highlights the dialectic between the Ego's functions and the drived dangers when facing the demands of the concrete reality.

## Introduction

Until Pavlov (1904 Nobel Laureate in Medicine, in recognition of his work on the physiology of digestion, through which knowledge on vital aspects of the subject have been transformed and enlarged), whenever a physiologist had a certain part of the brain removed, he also observed the animal's behavioral changes and he considered they were caused by the cerebral lesion. On the opposite side, the cerebral lesion produces an adjustment disorder to environment. The lesion produces a disruption of the bidirectional relationships between brain and the external environment. According to Pavlov, the integration function has no certain site. Any excitation arriving to the brain can form a conditional bond and can be associated with any activity.

The opposite theories evolved in time – the organicists looking for the organic support (structural and morphological) for any functional disorder, and, the functionalists not recognizing the organic support – have been disturbed for long time, solving the pathogeny and therapy for many diseases [3,7,9,1]. Pavlov and his disciples showed that there is a strong relationshipbetween organic and functional, by combining the removed part of the brain with the development of the conditional reflexes and establishing anatomical and physiological correlations on a materialistic base. The soviet researchers showed that in the fundamental processes – excitation and inhibition sitting at the base of the superior nervous activity – a wide range of enzimatical and chemical changes occur in nervous fibers, synapses and neurons. Here lies the essence of organic changing into functional. For example, an intense and long–term inhibition process can produce intense physical and chemical changes of the morphological support, which in time can result in structural alterations. In the proximity of a small brain lesion, many functional changes occur, that can explain the reversibility of an apparent irreversible status.

The nervous system functional status, especially for grey matter, represents a continuous image of the relationships between the nervous system and the external and internal environment. The functional status changes into an organical status when some changes occur, with respect to external or internal environment, so, the functional disorders are enhanced and this enhancement produces irreversible organically structural changes in the nervous system. The brain functionality has been recorded since 1928, when Berger invented the electro–encephalograph; to better understand the way reflexes described by Pavlov are produced. More and more sensitive, the EEG accurately describes everything that appears in the brain, but the difficulties in translating electrical language into physiological language remain.

Controversies about the cerebral hemispheres functions have existed since the past century and they create two positions: anti–localization and localization [7,1,6]. Anti–localizationists (P. Flourens, Goltz, K. Lashley etc.) sustained that the psychic functions cannot be localized and learning does not depend on certain zones, but on the brain as it; the disorders produced by brain lesions do not depend on the lesion's site, but on how much of the brain is disrupted [1]. The extreme localizationists (F. Gall) sustain that every function corresponds to certain parts of the cortex.

Nowadays, this controversy is solved by the dynamic localization concept; according to this concept, the complex functions are based on the relationships between many structures evolving during human existence. The functional relationships between these structures are the base of specific behavioral acting, activated according to external environmental significance and to internal motivations. A prompt and consistent switch will produce an adequate and efficient adjustment. Thus, an optimal behavior needs not only a functional differentiation and specialization, but also a switch from one neuronal frame to another when one is performing a programmed, correlated and integrated activity.

 ‘The brain appears to us as the most complex programmed system among all known real systems. Some of the programs that are directing its functioning are genetically conditioned; they are directly related to fulfilling the primary biological functions and to appropriate motivations. At the human level, the amount of inborn programs is limited and insufficient to assure the adjustment to the complex environment conditions. Thus, most of the programs are acquired, they are developed and they become operative through learning, based on conditioning mechanisms and laws: signalizing, association, reinforcement, correcting feedback, consolidation, automatization, selective generalization, etc.’[1]

The essential stimulus in the development of brain's integrative functions is information. The highest levels of information integration are the cognitive processes. The human psychic is, ontologically speaking, pure information. One can say that the psychic is a brain's function (accomplished as information's receiving–processing–interpretation–encoding) and information (constituted through successive processing and integrations in distinct entities named psychic processes and states) is delivered by the various categories of mechanical, physical, chemical and socio–cultural signals, acting from outside onto the individual's receptors.

The presence and the individuality of the psychic components are emphasized only through behavioral acts. In the early stages of psychogenesis, the external factors are dominant, and in the later ones, when the cognitive, motivational, instrumental, and regulatory structures are increasingly consolidated and autonomous, the internal organization is dominant. ‘With respect to behavior, one can conclude that the way the psychic develops is by going from external determination to self–determination, based on analysis–assessment–decision–correction’ [6]

In fate analysis, the psyche is defined as the life's process in its way to freedom. Leopold Szondi fought for the probabilistic vision, managing to overcome the Freudian causal vision. From the ‘mental stuff’ perspective we can rather explain it as an option (as an electoral process) applied to the existent possibilities which are potential figures in the familial inheritance of the individual. Ancestral figures have a strong impact on the human face and constitution, which expresses the optional concentration made by the genetic hazard.

The Szondian way of thinking is a probabilistic one: the individual comes with a multitude of existential virtualities that are his inheritance. From all the ways of living, that his ancestors had already lived, in the individual life, due to an environmental factor, but mostly because of these embryonic choices, an internal–external dialectic dialogue takes place. The familial unconscious function is made–up by the language of forced choice (obligation, forcing, factum), and, the ‘Pontifex Oppositorum’ Ego function is to transcend, to integrate and to participate in building bridges between the conscious and the unconscious opposites. From the very beginning of the contact with the reality (from the birth), the individual confronts with himself until he realizes, as a Superego, all the educational influences coming from the outside. Participation with the others is essential; the one with the nature and with God conducts to love another person, to communication and to faith.

The path of life does not have a final destination; this is made through the choices in: love (marriage), friendship (social ideal), profession (vocation as a base of the career), disease (specific somatic affections) and death (a certain way of dying). The forum, which has the power to choose a free destiny instead of a forced one, is Pontifex–Ego, namely the Ego functions that outrun the contradictions that come into the consciousness.

Through integration, the Ego must authoritatively dominate and guide all its elementary functions, which means that it will give diverse ancestral exigencies to the conscious mind, which will guide it in the world, into unconscious seeking (projection), and it will help in facing them with the reality. Whether one of these hereditary possibilities has a promise of a better destiny, the Ego approves, incorporates (introjections) or disapproves (denial) the forced destiny lived until present.

Through transcendence, the Ego needs the power to transcend to the spirit. It must try to establish a connection with a transpersonal idea (humanity, art, science, religion) and to activate the faith function common for all the individuals.

The spiritual participation requires the power to connect to the superior idea from the Ego, which means the power of participation.

The Ego receives the power and the strength to deny its forced destiny, to freely choose between the family's existential possibilities given to the conscious mind and its own human living destiny, from these functions.

Therefore, the Ego is the power assigner, the organiser and the administrator of the complementary coexistence and totally cooperation for the opposite's poles of conscious and unconscious soul, the bridge with the power to overrun the soul opposites. The Ego birth is equally the birth of the human soul being, first, the birth of the human existence in opposition with the animal existence.  

From the genetic point of view, from the base, the Ego is a derivative of an older psychic organisation – the Self (Freud described this, together with the Ego and the Super–Ego, as being the primary and stabile component of the personality, a source of the whole driving energy). The Ego has the function to serve as an intermediary between the instinctual claims of the Self and the exigencies of the external world, which is discovered through the perceptive system. This system also informs about the internal processes. Through its strong relationship with the motor system, the Ego modulates the ways of discharging the instinctual needs, which have an inner origin. 

The main responsibility of the Ego is to establish a coherent organisation of the personality, summing several types of conflicts into a result which will be satisfactory until a certain point the Self's basic demands, trying to avoid the conflicts with the external world or with the Super–Ego's moral demands. The means to realize this compromise are the identification and repression mechanisms. Through identification, the libido's basic assimilated object is incorporated in the Ego, desexualized and therefore no longer needed as an exterior object. So, the original drives from the Self are attached to the Ego, becoming more rational and easier to be satisfied, avoiding all the limits enforced by the reality or the by the Super–Ego.

The Ego vector expresses the dynamic force of the ‘instinctual’ drives, namely the manner they express themselves in a symbolic form to the conscious (p factor) and the way they are integrated in this coherent organization of the psychic life (k factor).

In ‘Trebpathologie Erster Band (Teil B., Wien, 1977, p.495), L. Szondi says: ‘The more the neurotic defense mechanisms are used by an epileptic (inhibition, repression, alienation), the more likely is the diagnostic of hystero–epilepsy. On the contrary, one must take into consideration organic epilepsy when the infantile Ego profiles dominate (projection, introprojection)’.

**Objective**: Using an extended pathology, the search correlates the results of the szondian testology with the medical ones in order to surprise the existing association between the Ego's mechanisms and the organic lesions.

**Context**:  ‘Bagdasar–Arseni’ Clinical Emergency Hospital, Neurosurgery Department, Bucharest, Romania.

## Clinical material and method

### Subjects

In the period of January 2000 – December 2004, 400 subjects were selected and were psychologically examined by using the Szondi technique and test. All of these patients were hospitalized in the neurosurgery clinics for specific pathologies such as intracranial expansive processes, with cerebral compression phenomena, due to tumoral, cystic or vascular cause. The following techniques of medical imagery were used as investigative methods: computer – tomography (CTscan), magnetic resonance (MRI) and the computerized electroencephalogram (EEG) used for the neurosurgical protocol. The patients' exclusion criteria were the traumatic causes of the present medical condition and the personal history of physical trauma (TBI or other types of surgical interventions). According to the existence or inexistence of the organic lesion, as shown by the investigations, the selected patients were grouped into two lots, which, through the resulted data, constituted the object of the statistic analyses.

### Procedure

The first approach of the patients made in the neurosurgical investigation phase in order to establish the diagnosis, on the condition that the psychic functions could allow the examination in an efficient collaboration. The emergency cases with brain– or vascular–related compression were examined post surgery, when the patient's mood allowed this. The working technique meant the administration of the Szondi test at the beginning and at the end of the session, two profiles were obtained in one hour; between these two administrations, the clinical interview was made in order to find out the history of the patient and obtain the data about the family. The next profiles were made during the next days, until the patient was discharged.

### Data analysis

The data obtained for each patient constituted in dependent variables (szondian profiles, reasons for hospitalization, period of onset) and independent variables (research investigation) in a socio–familial–professional personal context represented by the variables, contained the factual data (sex, age, family origin, personal family, education, profession, occupation). The statistical process shows, in a descriptive manner, the demographic aspects, central tendencies and the important correlations between variables. The tests of representation were used at an inferential level, in order to verify whether the results were extending to the whole population.

## Results presentation

### Demographic data

The studied group contains 400 patients, 47% men and 53% women, average age is 30.72 years old (std.dev.11.47). The educational level is medium (high school +/– post–high school studies, 31%), most of them working in technical fields (24%), mostly coming from equilibrated families, without special problems (70%) from urban environment (82%). Most of them were married (51%) at the time of the research, 29% were unemployed and 17% were out of profession (de–professionalized). 

The onset of the illness represents the reason for hospitalization for 40% of them, expressed either by coming in the emergency room or in a period of at most 3 months before, presenting grand–mal crisis (14%) and headaches or dizziness/confusions (10%). For 53% the symptoms were daily or permanent without relation with the external events. 

After the medical investigations, 56% had a neurosurgical diagnosis and 36% a neurological diagnosis; for the rest (8%) a psychiatric diagnosis was established.

- EEG signals were normal for 46%, focal for 10% +/– tendency to irradiation for 15% / 12%.
- A normal MRI was found in 52% of the patients and suggested tumors (11%), vascular diseases (18%), cystic conditions (9%) or ICH syndrome 10%.
- The lesions were found in 49% of the patients and 19% of them underwent operation.
- A medication treatment was administrated to 96%, including the operated persons.

Comparing the two groups, I have found the next characteristics presented in Table 1 below. The differences are for sex (men are dominant in the lesion group), education (medium), profession (technical field / unemployed in the no lesion group), occupation (without / pupil, student), marital status (married / unmarried), hospitalization reasons (headache, dizziness, confusion, nausea, vomiting / grand mal crisis), EEG (irradiation / normal), MRI (vascular / normal).

**Table 1:** Comparative demographics

characteristics	Lesion	No lesion
	N	mean/mode	DS /variance	freq	Percent	N	mean/mode	DS /variance	freq	Percent
age	196	33.76	10.34			204	27.80	11.77		
sex	196	male	0.23	100	51%	204	female	0.25	116	56.9%
education	196	medium	1.27	68	34.7%	204	studding	1.66	72	35.3%
profession	196	technical	37.92	52	26.5%	204	jobless/without	4.25	72	35.3%
occupation	196	without	1.95	56	28.6%	204	pupil, student	1.95	60	29.4%
residence	196	urban	0.17	160	78.4%	204	urban	0.11	168	85.7%
origin family	196	equilibrate		160	81.6%	204	equilibrate		120	58.8%
marital status	196	married	0.49	132	67.3%	204	unmarried		116	56.9%
debut	196	admitted	3.02	116	59.2	204	admitted	5.73	44	21.6%
evens association	196	no	0.23	128	65.3%	204	no	0.25	116	43.1%
admitted reasons	196	headaches, dizziness, confusion, nausea, vomiting	67313806	32	16.3%	204	grad-mal crisis	4.231^E^+10	40	19.6%
symptoms frequency	196	daily or permanent	37.42	112	57.1%	204	daily or permanent	131.73	100	49%
EEG	196	no irradiation	186.27	56	28.6%	204	normal	95.30	152	74.5%
MRI	196	vascular	65.43	72	36.7%	204	normal	0.00	204	100%
diagnosis	196	neurosurgical	2.89	192	98%	204	neurological	39.86	144	70.6%
surgery	196	no	0.23	124	63.3%	204	no	0.00	204	100%
treatment	196	medication	0.00	196	100%	204	medication	7.26^E^–02	188	92.2%

Through nonparametric tests, binominal and chi square, I have verified the representativeness of the two groups, a strong statistic meaning sustained (Table 2and Table 3).

**Table 2:** Binomial test's results

Binomial Test		Category	N	Observed Prop.	Test Prop.	Asymp. Sig. (1–tailed)
lesion	Group 1	1 yes	196	0.49	0.33	0.000
	Group 2	2 no	204	0.51		
	Total		400	1.00		

**Table 3:** Chi–square test's results

	debut	diagnosis	education	EEG	crisis freq.	Marital status	Admitted reasons	Occup.	Profess.	MRI
Chi–Square	312.320	492.400	59.600	282.080	990.400	477.200	623.840	29.200	244.000	786.182
df	7	4	4	5	9	4	53	4	9	8
Asymp. Sig.	0.000	0.000	0.000	0.000	0.000	0.000	0.000	0.000	0.000	0.000
	origin family	residence	surgery							
Chi–Square	2837.119	174.240	153.760							
df	15	1	1							
Asymp. Sig.	0.000	0.000	0.000							

Concerning the drive vectors, the two groups are represented in Table 4, built–up on course database stated in frequency and percentage.

**Table 4:** Descriptive profiles

Vectors	Sexuality		Paroxismality		Sch The Ego		Contact	
Profiles	lesion	no lesion	lesion	no lesion	lesion	no lesion	lesion	no lesion
0 0 disintegrated	0	4 (2%)	4 (2%)	12 (5.9%)	8(4.1%)	0	12(6.1%)	8(3.9%)
0 +possessed	4(2%)	8(11.8%)	0	0	8(4.1%)	12(5.9%)	36(18.4%)	36(17.6%)
0 – archaic	12(6.1%)	0	20(10.2)	24(11.8%)	20(10.2%)	8 (3.9%)	32(16.3%)	12(5.9%)
0 +/– feminine	0	12	0	4 (2%)	0	0	4(2%)	4 (2%)
– 0 juvenile	4 (2%)	4 (2%)	28 (14.3%)	4 (2%)	36 (18.4%)	24 (11.8%)	0	4
– + inhibited	8 (4.1%)	16 (7.8%)	8 (4.1%)	4 (2%)	44 (22.4%)	40 (19.6%)	12 (6.1%)	20 (9.8%)
– – adaptive	8 (4.1%)	4 (2%)	28 (14.3)	32 (15.7%)	16 (8.2)	40 (19.6%)	4 (2%)	4 (2%)
– +/– alienated	4 (2%)	12 (5.9%)	4 (2%)	16 (7.8%)	8 (4.1%)	4 (2%)	0	4 (2%)
+0 autistic	16 (8.2%)	24 (11.8)	20 (10.2)	8 (3.9%)	8 (4.1%)	12 (5.9%)	8 (4.1%)	32 (15.7)
+ + narcissist	48 (24.5%)	28 (13.7%)	4 (2%)	4 (2%)	8 (4.1%)	0	32 (16.3%)	16
+ +/– introjectant	24 (12.2%)	32 15.7%)	0	4 (2%)	0	0	20 (10.2)	8 (3.9)
+ – irrational	24 (12.2%)	36 (17.6%)	56 (28.6%)	64 (31.4%)	24 (12.2%)	20 (9.8%)	24 (12.2%)	28 (13.7%)
+/– 0 identified	16 (8.2%)	0	8 (4.1%)	12 (5.9%)	4 (2%)	20 (9.8%)	4 (2%)	8 (3.9%)
+/– +compulsive	4 (2%)	12 (5.9%)	4 (2%)	0	0	12 (12.2%)	0	12 (5.9%)
+/– –fugitive	16 (8.2%)	8 (4.1%)	16 (8.2%)	20 (9.8%)	8 (4.1%)	8 (3.9%)	0	0
+/– +/– integrated	8 (11.8%)	12 (5.9%)	0	0	0	0	0	0

## Statistical Correlations

There are correlated dependent variables in the study, represented by the Ego profiles and the patients' symptoms, with the independent variable, the injury, expressed through medical data constituted out of the investigations' results (MRI, EEG, and CTscan). Statistical tests were used for this purpose, adapted to the level pertaining to the variable, prevalent nominal and ordinal. Especially statistical meaningful correlations were selected and interpreted, between Sch vector and the independent variable.

The archaic Sch [0 –] is in inversed proportional relationship with the reasons of hospitalization (Spearman rho = –.412, Somers'd = –0.667, p<0.05) orienting to the grand–mal crisis' association with the headache (when [0 –!] appears), confusion, dizziness, nausea, vomiting and paresis (Chi square =0.000, p<0.05). The surgical intervention is less probable in patients with this profile (rho=0.645, p<0.001; Somers’d = 0.833, p<0.05), being presumed if [0 –!] it is registered (Chi square = 0.003, p<0.05).  The probability for this profile (contingency coefficient = 0.707, p<0.001) to be associated with the MRI results, suggesting vascular or cystic nature of intracranial compression, less probable tumoral–for which [0 –!] (Chi square =0 .000, p<0.05) appears.

The inhibited Sch [– +] orientates to an earlier debut (childhood, adolescence or later) and the vector straining [– +!!] suggests a closest debut (under 1 year). Sch straining [– ! +] grows the debut's probability as a reason of hospitalization (Chi square = 0.000, p<0.05). In a weak relationship with the lesion (rho = 0.272, Somers'd =0 .273, p<0.05), it is assumed to be localized in those patients with brain injury [–! +]. For [– +!] and [– +!!] it grows the probability that the patient does not have brain injury.

Hospitalization reasons could be influenced by this profile (81% according to Uncertainty Coefficient =0.809, p<0.05) to grand–mal crisis and headache, dizziness and concomitant with vector [– +!] and [– +!!] straining, the crisis is more likely of the petit–mal type (Chi square = 0.000, p<0.05). 

Surgical intervention lowers the probability of appearance of this profile (Chi square = 0.000, p<0.05; rho = –0.481, p<0.05). The MRI result is mostly negative for those who have this mechanism (Chi square =0.008, p<0.05; rho = –0.222, p<0.05).

The adaptive Sch [– –], in relationship with debut, can be met more frequently (Somers'd= –0.500, p<0.001) in those who are hospitalized on short term after the symptoms' debut, mostly at 3 months (rho=–0.413, p<0.05; Chi square = 0.001, p<0.05). The hospitalization reasons are, probably, headache, dizziness and petit–mal as well as grand–mal type crisis (rho = 0.502, p<0.05; Chi square = 0.000, p<0.05). 

EEG is prevalently normal, the irradiation tendency appears in those with Sch [–! –!] and, when Sch [– –!], the focalization is ascertained (rho = 0.396, p<0.05; Chi square = 0.000, p<0.05). The MRI, CTscan results have a tendency towards normality and the neurosurgical intervention is less probable in the patients with this profile (contingency coefficient = 0.484, p<0.001; Chi square = 0.000, p<0.05).

The narcissist Sch [+ +] correlates inversely proportional with the debut (Somers'd = – 1.000; rho = –1.000, p<0.001) suggesting that, when [+! +], it can be 1 year behind and when [+ +!], the debut is the reason of the hospitalization (Chi square = 0.014, p<0.05). 

The hospitalization reasons are headache, nausea, vomiting and loss of consciousness if [+! +], but with normal EEG; and for [+ +!], only with dizziness and confusion, the EEG records have an irradiation tendency (rho =1.000, Chi square = 0.014, p<0.05). In relation with MRI and CTscan results, [+! +] suggest a brain edema determined by a cyst, and [+ +!]  orientates towards vascular pathology (rho = 1.000, Chi square=0.014, p<0.05).

The identified Sch [+/– 0] orientates the debut towards a hospitalization reason or lasts for at least one year (Lambda 1.000, p<0.001; Chi square <0 .001, p<0.05); the possibility of an older debut, 3 years at the most, appears if [+/–! 0] is registered. It is in relationship with crisis frequency (rho =0.707, p=0.001; Chi square <0.001, p<0.05) meaning that it determines the daily crisis and can launch more crisis a day (prediction valid for 100% according to Uncertainty Coefficient = 1.000, p <0.001).

The fugitive Sch [+/– –] indicates the debut in childhood, adolescence or a later onset. It can be even newer, up to one year, when [+/–! –] (Lambda =1.000; Somers'd =1.000; rho =0.775, p =0.001; Chi square =0.001, p<0.05). It orientates prevalently to neurosurgical diagnosis but it can associate the neurological diagnosis with a psychiatrically one when [+/– –!] (Lambda =1.000, Somers'd =1.000, rho =0.816, p<0.001; Chi square <0.001, p<0.05). 

In association with the EEG recording (Somers'd =0.667, p<0.05; rho=0.544, p<0.05; Chi square <0.025, p<0.05) which distinguishes the focalization with irradiation tendency, the profile becomes [+/– –!] in a crisis frequency more than once a day (Somers'd=0.667, p<0.05; rho=0.544, p<0.05). The MRI and CTscan results distinguish vascular pathology and brain edema in relationship with [+/– –] and normality in relationship with [+/– –!] (rho = –0.544, p<0.05; Chi square <0.032, p<0.05).

The possessed Sch [0 +] the onset of symptoms, in relationship with this type of profile, is an old one, from childhood or adolescence, anyway, not closer than three years. When recording Sch [0 +!] a later onset is possible (100% according to Uncertainty Coefficient =1.000, p<0.001, Chi square <0.001, p<0.05). The diagnosis is prevalently neurological with neurosurgical possibility which determines [0 +!] (Lambda = 0.500, p <0.001; rho = –0.612, p<0.05; Chi square <0.001, p<0.05). It is more probable that the EEG recording is normal (rho = 0.612, p<0.05) and the crisis’ frequency often daily, once or more than once (Somers'd = –1.000, p<0.05; rho = –0.725, p<0.001; Chi square <0 .001, p<0.05). This profile is more frequent in the patients without injury (rho = –0.612, p<0.05, Chi square <0.001, p<0.05) and, if a cystic origin vascular formation is distinguished by the CTscan, than [0 +!] can appear (Somers'd =0.727, p<0.001; rho = 0.791, p<0.001; Chi square <0.001, p<0.05). Associated with neurosurgical intervention, the profile is more likely to be met in patients with no surgical indication; in a lesser extent, in the case of intervention, the profile becomes [0 +!] (rho =1.000, p<0.001; Chi square <0.001, p<0.05)

## Discussions

The present study (in a quantitative analysis, only) is part of a vast research concerning the dialectical relationship of the Ego vector and the other drives vectors into cerebral suffering lived by the human being as an alternative to efficient adaptation. The nexus reveals the correlations that exist between the other drive vectors and the lesion, as well as the correlation of the Ego's mechanisms with the other vectors' profiles in the lesion's presence (quantitative analysis, only). The final goal of the research is a destiny–analytical interpretation based on a qualitative analysis that describes the Ego function as intermediary of the instinctual Self's demands and the external reality exigencies.

The mind's double dependence – as a determinant factor of the outer world and of the brain as a mechanism – leads to the appearance of vast inter–individual differences registered and imposed to the mind – brain relationship, in order to be translated as a method of mutual dynamic incorporation of two hyper complex structures.

L. Szondi said, ‘As it is impossible to typify a human being without the Nervous System functions, it is, as well, impossible to typify him without the destiny system. Of course, we cannot see that system in an anatomical–topographical way, as well as we cannot discover his anatomical–pathological disturbances post mortem. Today it is possible to examine the psychological functions and the pathological disturbances of the destiny's system, with the help of the specific clinical methods.’ (‘Liberte et contrainte dans le destin des individu’, 1975, p.30)

We have to consider both the direct dependence of the psychic organization by brain organization and the reversed dependence of functional organization of the brain, by the psychic organization (psycho–physiological adjustable influence). It is necessary that the casuistic individual analysis is correlated with that of a statistic representative, to objectively determine the relationship between random and law. The analysis of the psycho–pathological effects caused by the brain focal injuries seeks to establish the prior normal function too, before the decay, for exact evaluation of the deficit dimension. The reconsideration of the relationship between the psychic and the brain appears as necessary, in order to be evaluated not only for the time being, but also in a dynamic way too, from a genetic–evolving perspective.

As part of a super–orderly integrative system, the state of a concrete psychic function will essentially depend on the state of the other components of the system. The functional organization's mechanisms of different particular psychic processes itself is based on systemic integrative principles. In the inner part of any particular mechanism a differentiation and a functional division takes place, the different structures or the systems that compose it realize certain sides or links of the integral psychic act.

The human brain realizes a socio–cultural form of psychic, not automatically but conditioned, because of the interaction and communication between man and the socio–cultural medium. The psychic appears and develops based on extraction, processing, hoarding and using the information contained in the signals emitted from these sources; just as obvious, the nervous system appears (the brain) as an organ (mechanism) of the psychic. In order to realize all the available potentials and for the competence to be transformed in performance, the contact and double sense communication relationship settlement are necessary – from interior to exterior and from exterior to interior. 

## Conclusions

1. Cerebral injury was identified in 49% of the patients and 56% had a neurosurgical diagnosis, on a tumoral, cystic, vascular pathology and hydrocephaly. Neurosurgical intervention indicated for 19% of the lesioned.
2. There are statistical significant differences, between the two groups constituted round the lesion (as two human beings – (healthy – non–lesioned and lesioned one), in the whole representative population.
3. Married men, with the average age of 33.76 years old and medium studies, mostly, constitute the group with organic lesions, which have professions in the technical field but have no work place. They were hospitalized for headache, dizziness, confusion, nausea and vomiting. The computerized EEG recordings had no tendency to irradiation and the MRI results suggested a vascular pathology. In a percentage hierarchy, the types of Ego profiles encountered in this group are: the juvenile [– 0] 18.4%; the inhibited [– +] 16.3%; the irrational [+ –] 12.2%; the archaic[0 –] 8.2%; the adaptive [– –] 6.1%; the fugitive [+/–  –] 4.1%; the alienated [– +/–] 4.1%; the disintegrated [00] 4.1%; the autistic [+ 0] 4.1%; the narcissist [+ +] 2%; the identified [+/– 0] 2%;  the  possessed [0 +] 2%. There is no choice for the integrated [+/– +/–]; the compulsive [+/– +]; the introjectant [+ +/–]; the feminine [0 +/–].
4. The group without the organic lesion is mostly constituted of women with the average age of 27.80 years old, unmarried, with medium studies, non professionalized, with unfinished studies–pupils or students. The reasons of the hospitalization are the grand–mal crisis for which the EEG recordings are normal and the MRI suggests normality. In this group, the types of Ego profiles encountered are the adaptive [– –] 15.7%; the juvenile [– 0] 11.8%; the inhibited [– +] 9.8%; the irrational [+ –] 9.8%; the identified [+/– 0] 7.8%; the autistic [+ 0] 5.9%. No choice for: the feminine [0 +/–]; the introjectant [+ +/–]; the integrated [+/– +/–]; the disintegrated [0 0]; the narcissist [+ +];
5. Statistically meaningful there are 7 types of Ego profiles,  in relation with the lesion: the archaic ego [0 –], the inhibited ego [– +], the adaptive ego [– –], the narcissist ego [+ +], the identified ego [+/– 0], the fugitive ego [+/– –] and the possessed ego [0 +]:

The archaic ego [o–] is more often seen in vascular or cystic cerebral pathology in which the grand–mal crisis is associated with other symptoms. The indication for surgery puts tension on the [p– !] factor.

The inhibited ego [– +] is predominantly seen in an older pathology and probably without cerebral lesion associated with grand–mal crisis. When the [k– !] factor is tensioned, the grand–mal crisis is associated with headache and dizziness, being the reason for hospitalization, and then the probability of a cerebral lesion grows. When the [p+!] or [p+!!] is observed, the crisis can be petit–mal and excludes the lesion; the onset period being of mostly one year.

The adaptive ego [– –] is predominantly seen when the onset is the reason for hospitalization and the suffering (visible through crisis of all types associated with headache, dizziness and confusion) is not older than 3 months. EEG predominantly normal suggests a tendency to irradiation when [k–! p–!] appears, and the existence of a focalization when only [p–!] occurs. The injury and the surgical indication are less probable for this profile.

The narcissist ego [+ +] is associated with a short–term onset, [+! +] for at most one year and [+ +!] rises the possibility of urgent hospitalization. The hospitalization reasons are headache, nausea, vomiting with the possibility to lose consciousness only if [k+!] is generated by a brain edema and a cystic lesion, with a normal EEG. If [p+!] is registered, the loss of consciousness is replaced with dizziness and confusion, EEG recording suggests irradiation tendency in a vascular–related injury.

The identified ego [+/– 0] is most frequently met in a recently suffering person (mostly up to one year) which requires hospitalization when [k+/–!]. It is supposed that the crisis exists, one or more/day.

The fugitive ego [+/– –] is prevalently met when the onset is old (childhood, adolescence) or later; [+/–! –] it is associated with onset in the last year. The diagnosis is prevalent neurosurgically, based on MRI or CTscan suggestions, of brain edema and vascular cause, and the EEG recordings suggest the normality.

The possessed ego [0 +] is more frequently met in an old suffering (childhood, adolescence), not closer than 3 years from present, with one or more crises a day, normal EEG and neurological diagnosis. If [0 +!] appears, it is associated with a later onset and with the possibility of a neurological diagnosis, based on CTscan suggestions of cystic formation with vascular origin and could have surgical indication.
